# Investigating the appraisal structure of spontaneous thoughts: evidence for differences among unexpected thought, involuntary autobiographical memories, and ruminative thought

**DOI:** 10.1007/s00426-023-01814-y

**Published:** 2023-03-31

**Authors:** Cati Poulos, Andre Zamani, David Pillemer, Michelle Leichtman, Kalina Christoff, Caitlin Mills

**Affiliations:** 1 Department of Psychology, University of New Hampshire, Durham, NH USA; 2 Department of Educational Psychology, University of Minnesota Twin Cities, Minneapolis, MN USA; 3 Department of Psychology, University of British Columbia, Vancouver, BC Canada; 4 Centre for Brain Health, University of British Columbia, Vancouver, BC Canada

## Abstract

Involuntary thinking occurs when mental states arise without intention. Such thoughts can take different forms, such as *involuntary autobiographical memories* (IAM), *ruminative* thoughts, and *unexpected* thoughts—all of which are popular areas of study, albeit in somewhat disparate literatures. Despite these mental states sharing a common thread of feeling involuntary in nature, it is nevertheless unclear what separates them phenomenologically. We conducted a set of exploratory and confirmatory experiments to elucidate the appraisal dimensions behind these forms of involuntary thought, with a particular interest in understanding the phenomenology behind unexpected thoughts that are predicted to violate expectations of both timing and content. Across two experiments, we found that unexpected thoughts had unique appraisal structures compared to the other two forms of involuntary thought: they were less identifiably cued, more surprising in content and timing, and offered new information (i.e., insight). We discuss how these distinctions support recent theories regarding the nature of unexpected thought and its relation to other forms of involuntary thinking, namely IAM and ruminative thought, which are the more commonly studied forms of involuntary thinking.

**Supplementary Information:**

The online version contains supplementary material available at 10.1007/s00426-023-01814-y.

Imagine that you have just gotten home from grocery shopping; while preparing to cook yourself dinner, you suddenly think about the lifespan of penguins. The thought is completely unexpected—you don’t harbor any meaningful connection to penguins, and you have certainly never considered their lifespan until now. This *unexpected* thought—one that feels like it arose “out of the blue” (Mills et al., 2021)—is likely a common experience for most of us, and it is considered to be a form of “spontaneous” or “involuntary” thinking (Berntsen, 2021; Christoff et al., 2016).^1^ Such unexpected thoughts seem to evoke surprise or unexpectedness in a manner that is distinct from other well-studied forms of involuntary thinking, however, such as ruminative thought and involuntary autobiographical memory (IAM; Berntsen, 2021; DuPre & Spreng, 2018; Mills et al., 2021); yet the experiential dimensions that distinguish them have not yet been explored. That is, what makes an unexpected thought seem unbidden, and what are its underlying qualities and perceived functionalities? Further, how do unexpected thoughts compare to IAM and ruminative thought, which are also involuntary by definition?

Borrowing approaches from the appraisal theories of emotion literature, the current set of studies explores if (and how) the appraisal dimensions of these three forms of involuntary thought differ, with a specific interest in determining whether unexpected thought is phenomenologically dissociable from IAM and ruminative thought. We begin by reviewing the current state of the literature on involuntary and spontaneous thinking, as well as the theoretical and empirical evidence that forms the basis for our predictions. We then describe an exploratory pilot study (Experiment 1) that attempts to clarify the similarities and differences among unexpected thought, IAM and ruminative thought, followed by a confirmatory study (Experiment 2) that focuses on replicating Experiment 1 using a revised set of instructions and includes a supplementary analysis incorporating machine learning.

## Current definitions and theoretical background

It is important to first describe how different forms of involuntary thinking are typically defined in the literature, as this may shed light on basic similarities and differences among the constructs. We emphasize up front that the focus of this research is on the momentary experiences of thought and not on trait-level patterns in thought.

With this in mind, *ruminative thoughts* are defined as thoughts that arise involuntarily and with repetitive, affectively-laden content that is often self-focused (Matthews & Wells, 2003; Nolen-Hoeksema, 2000; Nolen-Hoeksema et al., 2008; Treynor et al., 2003). For example, imagine that you accidentally trip and fall while walking to your office on campus, and you cannot help but replay the embarrassing moment over and over for the rest of the day. Such types of thought would be considered ruminative as they feature self-focused, affectively-laden content and arise without deliberate intention (or even despite deliberate attempts to prevent them). Notably, ruminative thought is a prominent feature of multiple clinical states, including anxiety, depression, and bipolar disorder (Kovács et al., 2020; Muris et al., 2005; Nolen-Hoeksema et al., 2008). However, rumination need not be negative in nature (Li et al., 2017)—for example, you may think all day about something positive that happened to you, like getting a promotion at work. In either case, rumination reflects an automatic (involuntary) process rather than a deliberate (voluntary) process, and it is often accompanied by affectively salient content. For this reason, some researchers have considered rumination to be a form of spontaneous thought, as the thoughts arise and unfold without deliberate control (DuPre & Spreng, 2018).

*Involuntary autobiographical memory (IAM)* is the involuntary retrieval of a memory that pertains to one’s personal experience, often evoked by cues in the environment (Berntsen, 1996, 2010, 2021). Berntsen (2010) proposed that IAM could be conceptualized as a means of remembering, building on previous work of Ebbinghaus (1964). For example, say you are walking past a flower stand, and you suddenly remember the time you went to a sunflower field with your best friend. While thoughts such as this may feel random or inconsequential, Berntsen (2010) proposed that IAM is both a ubiquitous and functional phenomenon emerging from the same episodic memory system as *voluntarily* recalled autobiographical memories. However, there have been mixed findings concerning the frequencies of IAMs and voluntarily recalled autobiographical memories—Rasmussen and Berntsen (2011) found that IAMs were more frequent when participants counted their thoughts over the course of a day using a mechanical counter. In contrast, Rasmussen et al. (2015) found no frequency differences between IAMs and voluntarily recalled autobiographical memories when recording memories via a smartphone. They did, however, find that voluntarily recalled memories were rated as more relevant to one’s current situation compared to IAMs (Rasmussen et al., 2015).

Finally, *unexpected thoughts* are mental states that appear to arise unbidden in the sense that one does not expect them to occur at the moment and their content is also surprising^2^ (Mills et al., 2021). Unexpected thoughts, as we define them here, have themselves been referred to as ‘spontaneous’ thoughts within the literature, perhaps because they may represent the paradigmatic case of spontaneity—arising involuntarily, and violating our expectations in terms of both timing and content (Mills et al., 2021); however, we adopt the term *unexpected* to better capture their unique properties and to avoid further confusion in terminology. We argue that what defines unexpected thoughts is simple—whether they *feel* surprising in nature. Based on this definition, it is worth preemptively discussing the possible overlap with other types of spontaneous thinking that have been identified in the literature. For example, task-unrelated thoughts (TUT), or thoughts that are unrelated to what one is currently doing (Smallwood & Schooler, 2015), need not be unexpected (e.g., many even argue that they can be intentional; Seli et al., 2016). Other forms of involuntary thoughts share closer overlap, including involuntary semantic memories (often termed *mind pops*), or the involuntary retrieval of an item from memory such as a word or image (Kvavilashvili & Mandler, 2004). Although mind “pops” can similarly be surprising, they are defined by their content being semantic in nature, whereas unexpected thoughts are content agnostic. Finally, spontaneous insight, which has traditionally been studied through the lens of creative problem solving, can also have a “feeling” of unexpectedness to it (Laukkonen et al., 2018, 2021). Some spontaneous insights may thus qualify as being unexpected, but the utility of unexpected thoughts is also not a necessary feature—some unexpected thoughts may be rather useless. Thus, while these forms of ‘spontaneous’ thought may be a type of unexpected thought, this may not always be the case.

One clear similarity among unexpected thought, IAM, and ruminative thought is their involuntary (or non-volitional) nature. In the words of Christoff et al. (2016), they should all arise with very low deliberate constraints—meaning they were not voluntarily initiated through cognitive control. A possible difference among these three forms of thought, however, is how easily their occurrence may be attributed to underlying causes. Specifically, rumination and IAM are more likely to have identifiable traces that are instantiated through internally or externally salient cues.

For ruminative thoughts, cues would include latent self-relevant concerns that manifest in affective and repetitive thinking patterns (DuPre & Spreng, 2018), whereas IAM is more likely to have more concrete cues, such as perceiving sensory stimuli that have an obvious resemblance to the cued thought. Indeed, approximately 85% of IAMs have an identifiable cue (or set of cues; Ball & Little, 2006; Schlagman & Kvavilashvili, 2008). Additionally, IAMs are thought to induce a mood congruence effect, such that an individual’s mood at memory retrieval aligns with the affective valence of the thought content (Berntsen, 1996; Berntsen & Hall, 2004). This finding may be similar for ruminative thought as well; the affectively laden content of any ruminative thought may coincide with one’s ongoing positive and/or negative feelings. As such, mood congruence may evoke less surprise in terms of content for both ruminative thought and IAM. Thus, differences in causal attribution may contribute to the unique phenomenology associated with each of these kinds of involuntary thought.

In line with this perspective, Mills et al. (2021) recently argued that the phenomenological experience of spontaneity in one’s own stream of thought may be due to how unexpected or surprising the thought feels. They suggest that everyone likely has their own implicit statistical model of their thought stream, modelling the probability that certain thought topics will arise. People tend to think about certain topics with varying probabilities (e.g., thinking about food has a high occurrence probability, whereas thinking about running for president has a low occurrence probability for most people). This assumption of an implicit model allows for the possibility that thoughts can violate one’s statistical regularities in terms of their *timing* and/or *content*. We contend that such dimensions may, in part, account for the phenomenological differences between ruminative thoughts, IAM, and unexpected thoughts.

**Fig. 1:**
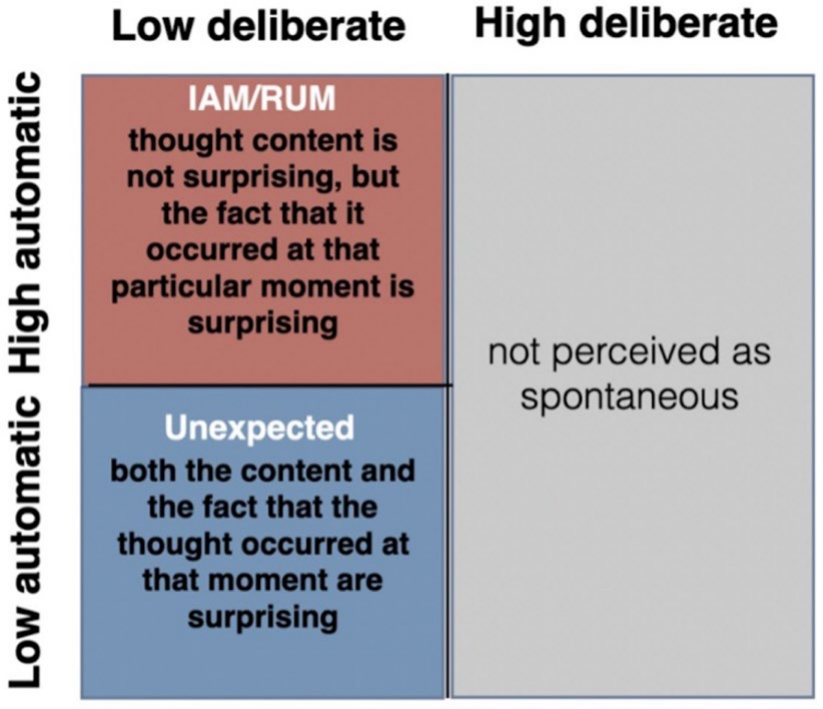
Proposed phenomenology of automatic and deliberate constraints on thought. Note: Rumination is abbreviated as RUM

Specifically, Mills et al. (2021) outline two forms of expectancy violation in one’s stream of thought. *Abrupt* transitions in thought refer to low-probability shifts based on timing alone, while the content of the thought itself is not low probability. That is, you do not expect the thought to arise at that moment, but you are not surprised at what the thought was about; these thoughts will be closely tied to salient current concerns, goals, and affective states, such that the content of these thoughts will be more highly automatically constrained by one’s internal or external environment (Christoff et al., 2016). As such, these transitions have a higher occurrence probability (Fig. 1). For example, you suddenly remember an item on your to-do list during an engaging discussion—the exact timing may be surprising, but the content itself is not.

In contrast, *wayward* transitions in thought refer to low-probability shifts based on *both* timing and content. You are not only surprised at when the thought occurred but also at what the content of the thought was about (Fig. 1). These thoughts are less consciously tied to one’s internal and external environment because cues are often not identified, meaning that automatic constraints are lower for such transitions. For example, thinking about the speed of hurricane winds while you are watching a documentary about the food industry would not only be surprising in timing, but also in content. Wayward transitions thus appear to closely align with our definition of unexpected thoughts, such that these thoughts should feel surprising in terms of timing *and* content due to their lack of identifiable cues. Ruminative thoughts and IAM, on the other hand, are likely to evoke less surprise based on their more easily attributable content, which is predicted to be more relevant to internally or externally salient stimuli; however, these claims have not been tested to date, and thus, we sought to test them here.

## An appraisal-based approach

The current set of experiments examines the appraisal dimensions behind the types of involuntary thought outlined here to determine if they exhibit unique appraisal patterns. Our approach is similar to those originally used in the *Appraisal Theories of Emotion*, where it is proposed that emotions are instantiated through the process of cognitively appraising or interpreting events (Ellsworth, 2013; Ellsworth & Scherer, 2003; Roseman & Smith, 2001). This allows for the same situation or event to elicit different emotions between or within individuals depending on their appraisal patterns (Moors et al., 2013).

Each emotion is thus distinguished according to relevant appraisals of a situation. Appraisal theorists expect that a number of dimensions contribute to an individual’s interpretation of an event, such as novelty and goal congruence, which then contribute to the subjective experience of an emotion such as fear, sadness, or guilt. Under this view, different emotions serve adaptive functions—for example, fear motivates one to escape from potential danger, while sadness enables one to seek support (Smith & Ellsworth, 1987). Here we propose that different forms of involuntary thinking may similarly serve different functions.

Historically, the emotion appraisal literature has examined the role of appraisals utilizing verbal self-reports of participants’ memories, although nonverbal techniques have also been used (Ellsworth & Scherer, 2003; Smith & Ellsworth, 1985). For example, participants may be asked to recall a number of experiences associated with a specific emotion (e.g., sadness) and then describe the experiences in as much detail as possible. This would then be followed by the participant rating the experiences on a variety of dimensions such as emotional valence, goal relevance, and novelty. The data are then used to construct the ‘causal determinants’ that together produce the experience of an emotion (Moors et al., 2013). We adopt these approaches in the current set of studies, while also including other dimensions that have been traditionally studied in the context of self-generated thought (defined as thoughts that are low in external constraints; Andrews-Hanna et al., 2013). For example, similar to the present set of studies, Andrews-Hanna et al. (2013) utilized the think-back paradigm to investigate how the contents of self-generated thoughts influence the perceived costs and benefits of such experiences (e.g., emotional impact). To do so, participants recalled 36 recent self-generated thoughts and rated these thoughts on a variety of phenomenological content dimensions to understand their characteristics. These included three general dimensions: level of construal (or abstract versus concrete processing), personal significance, and outlook. Several appraisals were measured for each dimension, such as the measurements of imagery within the construal dimension and emotional intensity within the personal significance dimension. As such, we adapted the dimensions from Andrews-Hanna et al. (2013)—specifically ones that were theoretically relevant to the thoughts investigated here based on prior research, such as dimensions of emotional valence and intensity, imagery, and value.

We begin by describing an initial pilot study (Experiment 1). The goals were two-fold: first, we tested if our predicted theoretical dissociations across the three types of involuntary thought had initial empirical traction. Second, we included a set of exploratory appraisal dimensions, derived from the affect and self-generated thought literature, that were included to inform a more targeted Experiment 2.

Related to our first goal, we made specific predictions about dimensions of identifiable cues, perceived spontaneity, and surprise in thought timing and content. Following the theories outlined above, we predicted that unexpected thought would have significantly fewer cues than IAM and ruminative thought. Second, we predicted that unexpected thought would be significantly more surprising in terms of both timing and content. Finally, we predicted that unexpected thought would feel significantly more spontaneous compared to IAM and ruminative thought. These basic comparisons allowed us to test, for the first time, if unexpected thought is phenomenologically dissociable from ruminative thought and IAM.

Related to the second goal, we were interested in identifying other dimensions on which these three forms of involuntary thinking might vary. This is particularly true for the types of content or perceived functionality that may come along with unexpected thoughts. That is, do these seemingly “random” thoughts have some benefits? To address this question, we included content dimensions from Andrews-Hanna et al. (2013) and the emotion appraisal literature as an exploratory set of dimensions; we did not make explicit predictions regarding their outcomes but included them based on their relevance to other related constructs such as mind wandering and affect.

Given the aforementioned two goals, and for readability purposes, we only focus on the results from our first goal (testing the empirical traction of our theoretical predictions). All exploratory results pertaining to the second goal are presented in the tables. In Experiment 2, these dimensions were then intentionally included and, as such, will be reported in more detail.

## Pilot study: experiment 1

### Method

All methods and procedures were approved by the IRB at the University of New Hampshire.

#### Participants

In total, 95 participants (83.16% female, 15.79% male, 1.05% non-binary) aged 18–42 years old (M = 19.33, SD = 2.55) completed Experiment 1. All participants were undergraduate students who participated for course credit using the online survey platform Qualtrics.

For the initial pilot study, we did not conduct an a priori power analysis as we did not have any effect sizes on which to base our analyses. Instead, we use the results from this initial study to look for initial patterns in the data and to guide our design of a replication.

#### Design

This study used a single-factor (3 levels: unexpected thought, IAM, ruminative thought) within-subjects design. The presentation of thought prompts, which instructed participants to recall a thought, was counterbalanced, such that for every 30 participants, the ordering of recall (unexpected thought, IAM, ruminative thought) was switched. The first 30 participants completed in the order ruminative thought, unexpected thought, IAM; the next 30 participants completed in the order unexpected thought, IAM, ruminative thought; and the final 35 participants completed in the order IAM, ruminative thought, unexpected thought. Participants answered appraisal questions for each thought immediately after typing out a description of the thought.

If a participant did not provide a response to a thought probe, this was coded as a failure and the corresponding ratings were removed from all analyses. Additionally, if a participant provided the same response to more than one thought prompt, only the first response was kept, and the rest were coded as failures and removed from the analyses. The failure rates for each thought type were: 1.05% (unexpected), 1.40% (IAM), and 1.40% (ruminative). In total, 844 thoughts/memories out of 855 were recalled (11 failures).

#### Materials

Participants were told they would be recalling and typing out three surprising thoughts, three involuntary autobiographical memories, and three ruminative thoughts that they had previously experienced, for a total of nine thoughts. Subtle changes in wording were used to indicate which type of thought participants were to recall. Participants were prompted to recall instances of IAM with “*Think back to a time when you had a specific memory involuntarily pop into your head*.” Ruminative thought was prompted with “*Think back to a time when you had a series of repetitive thoughts involuntarily pop into your head*”. Unexpected thought was prompted with “*Think back to a time when you had a thought that felt like it came out of the blue involuntarily pop into your head.*” All thought prompts used for Experiments 1 and 2 can be viewed in Table 1.

**Table 1:** Experiment 1 and 2 thought prompts

Thought type	Experiment 1 Prompts	Experiment 2 Prompts
Unexpected thought	Think back to a time when you had *a thought that felt like it came out of the blue* involuntarily pop into your head	Think back to a time when you had *an unexpected thought* involuntarily pop into your head
IAM	Think back to a time when you had *a specific memory* involuntarily pop into your head	Think back to a time when you had *a specific memory* involuntarily pop into your head
Ruminative thought	Think back to a time when you had *a series of repetitive thoughts* involuntarily pop into your head	Think back to a time when you had a *repetitive thought* involuntarily pop into your head

After typing out a description of a recalled thought, participants answered a series of questions pertaining to the appraisal of each thought. First, participants were qualitatively asked where they were or what they were doing when the thought occurred, followed by if they believed there was a specific cue that prompted the thought (answered by either yes or no). Next, participants were asked to rate how surprising the *content* of the thought was using a scale from 0 to 5, as well as how surprising the *timing* of the thought was using a scale from 0 to 5. Finally, as a manipulation check and for a “sanity check”, participants were then asked to rate how spontaneous the thought felt using a scale from 1 to 10.

After this, participants completed additional questions regarding our exploratory dimensions which were based on Andrews-Hanna et al. (2013) and included measures of the level of construal, personal significance, and outlook. Participants were finally directed to a questionnaire asking about the general valence of their thoughts, how they would personally describe a spontaneous thought, and how often they think they experience spontaneous thoughts. All questions are shown in Table 2.

**Table 2:** Experiment 1 and 2 appraisal dimensions, scales, and appraisal questions

Appraisal dimension	Exp 1 scale^a^	Exp 2 scale^a^	Question^b^
A priori dimensions			
Cue	0 = no 1 = yes	0 = no 1 = yes	Did you feel like there was a specific cue that prompted this *unexpected thought/repetitive thought/memory*?
Surprising in content	0–5	1–7	This *unexpected thought/repetitive thought/memory* was surprising because of its content
Surprising in timing	0–5	1–7	This *unexpected thought/repetitive thought/memory* was surprising because of its timing
Spontaneous (Exp 1)/unexpected (Exp 2)	1–10	1–7	How spontaneous/unexpected would you say this *unexpected thought/repetitive thought/memory* was?
Exploratory dimensions			
New information	0–5	1–7	At the time, I considered this *unexpected thought/repetitive thought/memory* to contain new information or a new perspective
Involuntary	0–5	1–7	I found this *unexpected thought/repetitive thought/memory* to be surprising because it involuntarily popped into my head
Task-unrelated	0–5	1–7	I found this *unexpected thought/repetitive thought/memory* to be surprising because it was unrelated to what I was currently doing
Thought-unrelated	0–5	1–7	I found this *unexpected thought/repetitive thought/memory* to be surprising because it was unrelated to what I was previously thinking about
Personally immoral	0–5	1–7	I found this *unexpected thought/repetitive thought/memory* to be surprising because the content of this *unexpected thought/repetitive thought/memory* contradicted personal morals or norms
Socially immoral	0–5	1–7	I found this *unexpected thought/repetitive thought/memory* surprising because the content of this *unexpected thought/repetitive thought/memory* contradicted societal morals or norms
Beneficial in time	0–4	1–7	At the time, this *unexpected thought/repetitive thought/memory* was… (very unbeneficial to very beneficial)
Beneficial now	0–4	1–7	When thinking back on it, this *unexpected thought/repetitive thought/memory* was… (very unbeneficial to very beneficial)
Value	0–5	1–7	This *unexpected thought/repetitive thought/memory* is/was of great value or importance to me
Solve problem	0–5	1–7	This *unexpected thought/repetitive thought/memory* helped me solve a problem
Goal	0–5	1–7	This *unexpected thought/repetitive thought/memory* involves/involved reaching a particular goal of mine
Self-relevance	0–5	1–7	This *unexpected thought/repetitive thought/memory* is/was highly self-relevant
Self-insight	0–5	1–7	This *unexpected thought/repetitive thought/memory* gave me new insight about myself
Others-insight	0–5	1–7	This *unexpected thought/repetitive thought/memory* gave me new insight about other people
Emotional valence^c^	1–10	N/A	My emotions pertaining to this *unexpected thought/repetitive thought/memory* are…(very negative to very positive)
Positive emotion^c^	N/A	1–7	My emotions pertaining to this *unexpected thought/repetitive thought/memory* are positive
Negative emotion^c^	N/A	1–7	My emotions pertaining to this *unexpected thought/repetitive thought/memory* are negative
Emotional intensity	1–10	1–7	The intensity of my emotions pertaining to this *unexpected thought/repetitive thought/memory* are… (no emotion to very intense emotions)
Mental image vividness	0–4	0–4	When I experienced this *unexpected thought/repetitive thought/memory*, my mental imagery was… (no imagery at all to perfectly clear)

^a^Lower scale values reflect the lower agreement, while upper scale values reflect the higher agreement

^b^In Experiment 1, instances of rumination were referred to as repetitive episodes, while in Experiment 2, instances of rumination were referred to as repetitive thoughts

^c^In Experiment 2, emotional valence was changed to include separate questions for positive and negative affect

#### Procedure

After granting informed consent, participants were prompted to recall and type descriptions of three instances of IAM, unexpected thought, and ruminative thought for a total of nine thoughts. After providing a typed description of each thought, participants answered a series of questions pertaining to the appraisal of each thought, including content variables from Andrews-Hanna et al. (2013) and the emotion appraisal literature. A full list of the questions and corresponding scales for each item can be found in Table 2. Lastly, participants completed a demographics questionnaire and were thoroughly debriefed on the purpose of the experiment.

#### Analytical approach

Due to the repeated measures nature of our data, all analyses were run using linear mixed-effects regression analyses using the *lme4* package in R (Bates et al., 2015). Thought condition and trial number were entered as fixed effects and participant was included as a random intercept in all models to account for individual differences in how participants respond to scaled questions. Unexpected thought was used as the reference group for all models, as a central aim of our studies was determining how unexpected thought specifically differs from IAM and ruminative thought. The one exception to the linear models was for the binary variable identifiable *cue* (yes = 1, no = 0), where we used logistic regression. Otherwise, the model setup was the exact same. In all cases, we first assessed the omnibus effect of the condition using the ‘car’ package in R (Fox & Weisberg, 2019) to assess if there was a main effect of the thought condition. If the main effect of condition was significant, we then conducted Tukey’s pairwise comparisons using the ‘emmeans’ package (Lenth et al., 2021) to compare unexpected thought, IAM, and ruminative thought.

In total, across the predicted and exploratory appraisal dimensions, we tested differences for 21 content variables in this pilot experiment. Given that we did not calculate power a priori, we carefully considered our alpha (*α*) level to avoid excessive Type 1 errors. For example, if we were to set *α* at 0.05, then we were almost guaranteed to make at least one Type I error. We thus decided a priori to set our *α* to 0.01 to minimize egregious Type 1 errors—attempting to strike a balance between chance results and the fact that we were not able to estimate effect sizes before collecting data. Again, we emphasize that we do not use this experiment to make strong claims; instead, we use this initial study to guide our replication design by considering effect sizes (Cohen’s *d*) and balancing the possibilities for both Type 1 and Type 2 errors.

### Results

To illustrate the types of recalls we collected, Table 3 includes examples of each type of thought reported by actual participants. All of our numeric data are available on OSF (https://osf.io/wdgcy/?view_only=3161fc311aaa4ad2891327a9d88bcf3c); full recall data are also available upon request.

**Table 3:** Examples of thought recalls

Unexpected thought	1. I questioned why humans do not consume acorns as they're extremely prevalent and other animals eat them. It was an extremely random thought 2. How exactly does the thought process of animals who have no language made of any diction, really work? What do their thoughts essentially take the form of? 3. What does it feel like to be color blind and not be able to see colors like other people?
IAM	1. I remembered the moment my grandmother told us they found something in her lung scan. It was after my brother's party. We were outside. It was a beautiful day, pre-COVID. Warm, sunny. There were no clouds in the sky. The chairs were spaced out and we were sitting in the front yard. All of a sudden, she blurted it out that they found something. She quickly went on that it was unknown, that it could be cancer, it could be aggressive cancer. She told us that if she died from it, she would want us to move on and not be too sad about it "because there are better things to do". I tried not to cry, I did not think about crying. I felt like I needed to let the information come and watch for my brother and my dad. Later, I think I cried 2. I was watching the TV show "Blown Away" which is a glass-blowing competition. They have fire torches and hazardous things everywhere. In one shot there were at least six unattended flames while people rushed through the room. I instantly was thrown back into my biomedical science lab in high school; a group left their tiny Bunsen burner flame unattended for maybe 30 s and she kicked them out of the lab for the rest of the day. So as I watched this TV show I was just in shock at the hazards and could only think of how mortified that teacher would be if she saw the show 3. The other day I was walking through campus and someone was talking to their friends and repeating the name of my ex-boyfriend. I heard it so clearly from so far. The memory of us on a hike with a few other people popped into my head. My mind grabbed me by the arm and brought me back a few years. I was able to perfectly picture the scene
Ruminative thought	1. One time I was really frustrated with my classwork and I could not stop thinking about how I must not be smart enough to finish school 2. I played soccer in high school. I was a defender and I honestly was not very skilled. My favorite thing to do was just kick as hard as I could and just send it down the field. We had a game under the lights at home against our biggest rival, and I was super nervous because the entire school was going to be there. I had been practicing shots on goal from half field, but never imagined I'd ever make one. But during that game I shot and scored. I could not stop thinking about how I could not believe I actually did it, let alone in that game. I was so shocked that all I could think was "wow, did I really just do that?" 3. Today, when I was leaving for class I remembered putting my computer in my bag, but the thought that I was forgetting it was repeating in my head. I checked twice because the thought kept persisting

Means, standard deviations, and Cohen’s d values are provided in Table 4; Chi-square values, b coefficients, and p-values are provided in Table 5. We note up front that we found a significant main effect of Condition (using an *α* = 0.01) for *all* 21 appraisal dimensions examined, with two exceptions: value (*p* = 0.030) and insight to others (*p* = 0.670) (Table 5). We refer readers to Table 5 for details of the main effects, and in the text below we focus on the pairwise comparisons for those analyses involving a priori predictions.

**Table 4:** Experiment 1 descriptive statistics and effect sizes

Appraisal dimension	Unexpected M (SD)	IAM M (SD)	Ruminative M (SD)	Cohen's *d* unexpected-IAM	Cohen's *d* unexpected-ruminative	Cohen's *d* IAM-ruminative
A priori dimensions						
Cue (proportion)	0.46 (0.33)	0.71 (0.29)	0.67 (0.32)	− 0.82*	− 0.65*	0.14
Surprising in content	2.98 (1.14)	2.07 (1.17)	1.72 (1.27)	0.79*	1.04*	0.28
Surprising in timing	2.80 (1.33)	2.32 (1.34)	1.58 (1.12)	0.36*	0.99*	0.60*
Spontaneous	7.04 (2.00)	5.89 (1.96)	4.94 (2.23)	0.58*	0.99*	0.46*
Exploratory dimensions						
New info	1.96 (1.27)	1.12 (1.15)	1.47 (1.23)	0.69*	0.39*	− 0.29
Involuntary	3.38 (1.19)	2.76 (1.25)	2.22 (1.41)	0.51*	0.89*	0.41*
Task-unrelated	2.96 (1.22)	2.20 (1.45)	1.95 (1.30)	0.57*	0.80*	0.18
Thought-unrelated	3.37 (1.28)	2.60 (1.32)	2.05 (1.32)	0.59*	1.01*	0.42*
Personally immoral	0.95 (1.03)	0.57 (0.78)	0.86 (1.03)	0.42*	0.09	− 0.32
Socially immoral	0.83 (1.03)	0.49 (0.73)	0.79 (0.99)	0.38*	0.04	− 0.34*
Beneficial in time	1.91 (0.96)	1.93 (0.82)	1.52 (1.05)	− 0.02	0.39*	0.44*
Beneficial now	1.96 (1.02)	2.04 (0.87)	1.49 (1.06)	− 0.09	0.44*	0.56*
Value	2.35 (1.25)	2.71 (1.26)	2.56 (1.45)	− 0.29	− 0.16	0.11
Solve problem	1.25 (1.19)	0.88 (1.06)	1.43 (1.22)	0.33	− 0.15	− 0.48*
Goal	1.28 (1.21)	1.01 (1.04)	1.72 (1.40)	0.24	− 0.34*	− 0.58*
Self-relevance	1.84 (1.24)	1.87 (1.33)	2.27 (1.40)	− 0.02	− 0.32*	− 0.30
Insight to others	1.08 (1.08)	1.15 (1.18)	1.17 (1.19)	− 0.06	− 0.08	− 0.02
Insight to self	1.44 (1.31)	1.22 (1.16)	1.71 (1.39)	0.18	− 0.20	− 0.39*
Emotional valence	5.82 (1.97)	5.78 (2.11)	4.13 (1.62)	0.02	0.93*	0.87*
Emotional intensity	5.39 (1.89)	6.06 (1.86)	6.06 (2.03)	− 0.35*	− 0.34*	0.00
Mental image vividness	2.03 (1.16)	2.81 (0.79)	1.95 (1.11)	− 0.79*	0.08	0.90*

*p < 0.01

**Table 5:** Experiment 1 Chi-squares, *b* coefficients, and *p* values

Appraisal dimension	Main effect (*Χ*^*2*^) (*p* value) *df* = 2	Pairwise comparisons (*b*) (*p* value)			
		Unexpected-IAM	Unexpected-ruminative	IAM-ruminative	Pattern^a^
A priori dimensions					
Cue	45.22 (*p* < 0.001)**	− 1.17 (*p* < 0.001)**	− 0.95 (*p* < 0.001)**	0.22 (*p* = 0.481)	UT < RUM = IAM
Surprising in content	89.56 (*p* < 0.001)**	0.93 (*p* < 0.001)**	1.26 (*p* < 0.001)**	0.33 (*p* = 0.047)	RUM = IAM < UT
Surprising in timing	77.66 (*p* < 0.001)**	0.48 (*p* = 0.002)*	1.22 (*p* < 0.001)**	0.73 (*p* < 0.001)**	RUM < IAM < UT
Spontaneous/unexpected	107.48 (*p* < 0.001)**	1.16 (*p* < 0.001)**	2.08 (*p* < 0.001)**	0.92 (*p* < 0.001)**	RUM < IAM < UT
Exploratory dimensions					
New info	42.77 (*p* < 0.001)**	0.84 (*p* < 0.001)**	0.49 (*p* < 0.001)**	− 0.35 (*p* = 0.019)	IAM = RUM < UT
Involuntary	98.13 (*p* < 0.001)**	0.64 (*p* < 0.001)**	1.16 (*p* < 0.001)**	0.52 (*p* < 0.001)**	RUM < IAM < UT
Task-unrelated	51.61 (*p* < 0.001)**	0.78 (*p* < 0.001)**	1.02 (*p* < 0.001)**	0.24 (*p* = 0.250)	RUM = IAM < UT
Thought-unrelated	96.17 (*p* < 0.001)**	0.79 (*p* < 0.001)**	1.32 (*p* < 0.001)**	0.52 (*p* < 0.001)**	RUM < IAM < UT
Personally immoral	14.47 (*p* < 0.001)**	0.39 (*p* < 0.001)**	0.10 (*p* = 0.596)	− 0.29 (*p* = 0.020)	IAM = RUM = UT
Socially immoral	14.45 (*p* < 0.001)**	0.34 (*p* = 0.001)**	0.05 (*p* = 0.850)	− 0.29 (*p* = 0.008)*	IAM < RUM = UT
Beneficial in time	24.38 (*p* < 0.001)**	− 0.02 (*p* = 0.965)	0.40 (*p* < 0.001)**	0.42 (*p* < 0.001)**	RUM < UT = IAM
Beneficial now	36.15 (*p* < 0.001)**	− 0.09 (*p* = 0.653)	0.46 (*p* < 0.001)**	0.55 (*p* < 0.001)**	RUM < UT = IAM
Value	7.03 (*p* = 0.030)	NA	NA	NA	NA
Solve problem	17.93 (*p* < 0.001)**	0.35 (*p* = 0.017)	− 0.18 (*p* = 0.332)	− 0.53 (*p* < 0.001)**	IAM = UT = RUM
Goal	25.76 (*p* < 0.001)**	0.27 (*p* = 0.131)	− 0.44 (*p* = 0.006)*	− 0.71 (*p* < 0.001)**	IAM = UT < RUM
Self-relevance	11.03 (*p* = 0.004)*	− 0.02 (*p* = 0.985)	− 0.41 (*p* = 0.009)*	− 0.39 (*p* = 0.015)	UT = IAM = RUM
Insight to others	0.80 (*p* = 0.670)	NA	NA	NA	NA
Insight to self	14.07 (*p* < 0.001)**	0.23 (*p* = 0.167)	− 0.25 (*p* = 0.127)	− 0.47 (*p* < 0.001)**	IAM = UT = RUM
Emotional valence	76.42 (*p* < 0.001)**	0.03 (*p* = 0.988)	1.67 (*p* < 0.001)**	1.64 (*p* < 0.001)**	RUM < IAM = UT
Emotional intensity	15.70 (*p* < 0.001)**	− 0.67 (*p* = 0.002)*	− 0.67 (*p* = 0.002)*	0.00 (*p* = 1.00)	UT < IAM = RUM
Mental image vividness	82.49 (*p* < 0.001)**	− 0.78 (*p* < 0.001)**	0.08 (*p* = 0.725)	0.86 (*p* < 0.001)**	RUM = UT < IAM

*p ≤ 0.01; **p ≤ 0.001

^a^*UT* = unexpected thought; *RUM* = ruminative thought

#### Number of cues

Pairwise comparisons indicated that unexpected thought had significantly fewer cues than both IAM and ruminative thought. There was no significant difference between IAM and ruminative thought. These results are in line with our prediction, given that IAM and ruminative thought are more likely to be cued from internally or externally salient information compared to unexpected thought (Ball & Little, 2006; Schlagman & Kvavilashvili, 2008).

#### Surprising in content

Unexpected thought had significantly more surprising content than both IAM and ruminative thought. There was no significant difference between IAM and ruminative thought. These results are in line with the idea that ruminative thought and IAM may be more representative of abrupt transitions in thought where the thought content is somewhat expected, particularly in comparison to unexpected thought.

#### Surprising in timing

Unexpected thought was rated as significantly more surprising in timing compared to IAM and ruminative thought. Further, IAM was significantly more surprising in timing than ruminative thought—perhaps because individuals come to expect their ruminations to be more probable thoughts in general.

#### Spontaneity

As a final test of how participants view spontaneity in their thought stream, participants reported that unexpected thought felt significantly more spontaneous than IAM and ruminative thoughts. Additionally, IAM felt significantly more spontaneous than ruminative thought. These results were also expected, as unexpected thought is more surprising in content and timing compared to IAM and ruminative thought due to a lack of identifiable cues, making it more likely to feel spontaneous.

### Discussion

The purpose of Experiment 1 was to gain preliminary insight into how three types of involuntary thought (unexpected thought, IAM, and ruminative thought) varied on several theoretically relevant appraisal dimensions. Supporting our hypotheses, we found that unexpected thought had significantly fewer cues and was more surprising in content and timing compared to IAM and ruminative thought. We also found that unexpected thoughts felt significantly more spontaneous than IAM and ruminative thoughts. These findings may be explained by the distinction between abrupt and wayward transitions in thought proposed by Mills et al. (2021). That is, while IAM and ruminative thought have a higher probability of occurring due to their reliance on implicit and explicit cues, unexpected thoughts are phenomenologically unbidden and surprising, such that they have a lower probability of occurring.

#### Exploratory analyses summary

Beyond our theoretical predictions, we also found that unexpected thought was more likely to feel involuntary, provide new information, be task-unrelated, be unrelated to the previous ongoing train of thought, and be less emotionally intense compared to IAM and ruminative thought. Providing new information is a particularly intriguing finding that aligns with the prediction that unexpected thoughts are likely to support novelty in one’s train of thought and relate to previously documented phenomena such as sudden insight (Topolinski & Reber, 2010). Additionally, the fact that unexpected thought is less intense emotionally is not surprising due to its independence from implicit and explicit cues such as affective state, current concerns, or goals.

These initial findings suggest that unexpected thought may be phenomenologically distinct from both IAM and ruminative thought. However, there are several possible limitations that may have influenced our conclusions from Experiment 1, which we attempted to address in a second experiment that served as a formal test of our predictions.

First, participants in Experiment 1 were instructed to appraise their thoughts directly after recall, and this may have biased their future recalls. For example, recalling a thought which is associated with a negative emotion may bias participants to recall subsequent thoughts that are also associated with a negative emotion. In Experiment 2, participants were thus instructed to type out all prompted thoughts *before* appraising them. Second, Experiment 1 used a single question to probe emotional valence, preventing the chance of identifying a thought that may be both positive and negative; to address this, we utilized two questions in Experiment 2: ratings of positive emotion and ratings of negative emotion. Third, we used the term ‘repetitive episodes’ for the ruminative thought appraisal items. This language may have carried an inherently negative connotation, potentially implying a more pervasive pattern of repetitive thinking. We thus used the cue ‘repetitive thoughts’ in Experiment 2 for the appraisal items to allow the possibility of identifying positive ruminative thoughts and to also ensure consistency between appraisal items. We also changed the rumination recall probe wording to ‘repetitive thought’ rather than ‘a series of repetitive thoughts’. Fourth, we prompted participants to recall unexpected thoughts using ‘*Think back to a time when you had**** a thought that felt like it came out of the blue**** involuntarily pop into your head’*. We changed this in Experiment 2 to ‘*Think back to a time when you had an ****unexpected thought**** involuntarily pop into your head.*’ (see Table 1) to provide a more concise definition. In addition, for the appraisal dimension of spontaneity, we changed the wording to reflect ‘unexpected’ in Experiment 2, given that spontaneity may be interpreted in different ways. This wording change also serves as a test of differences in “unexpectedness” across the three thought dimensions. Fifth, we modified all items in Experiment 2 to reflect a 1–7 scale (apart from mental image vividness) to provide consistency in responses and ensure findings were not an artifact of the way they were measured. Finally, as we did not run an a priori power analysis for this initial study due to a lack of effect sizes, we expanded our sample size in Experiment 2 to ensure adequate power was achieved based on the effect sizes from Experiment 1.

In addition to these methodological changes, we also incorporated the Autobiographical Memory Characteristics Questionnaire (AMCQ) (Boyacioglu & Akfirat, 2015), an established measure from the IAM literature, as a measure of convergent validity. The AMCQ was developed to assess a number of dimensions of IAM phenomenology, such as vividness, emotional intensity, and belief in accuracy. The AMCQ consists of 14 dimensions whose corresponding 63 items are answered on a scale from 1 to 7. Because the AMCQ was designed to examine memories and not qualities of general thoughts, we include these exploratory results in Online Resource 1.

## Experiment 2

### Method

All methods and procedures were approved by the IRB at the University of New Hampshire.

#### Participants

Experiment 1 revealed significant results with 95 people with a range of corresponding effect sizes (see Table 4). We thus increased our sample size to minimize any Type 1 or Type 2 errors that may have been present in Experiment 1. An a priori power analysis conducted in *R* using the ‘simr’ package (Green et al., 2019) revealed that a sample size of 396 was deemed to be a minimum viable sample size in order to detect a small to medium effect (*d* = 0.25) with at least 95% power (*α* = 0.001) in a repeated measures design. Note that we intentionally chose a very conservative alpha (*α* = 0.001; equivalent to a Bonferroni correction) given how many comparisons we made.

In total, we collected data from 421 participants (76.96% female, 21.85% male, 1.19% non-binary) aged 18–46 years old (M = 19.07, SD = 1.89) at the University of New Hampshire. All participants were undergraduate students who participated for course credit using Qualtrics, an online survey platform.

#### Design

This study was almost identical to Experiment 1 with a single-factor within-subjects design and counterbalanced presentation of thought prompts. A total of 177 participants completed the order IAM, ruminative thought, unexpected thought; 127 participants completed the order ruminative thought, unexpected thought, IAM; and 117 participants completed the order unexpected thought, IAM, ruminative thought.^3^ The major change from Experiment 1 to Experiment 2 is that appraisal questionnaires for each thought were completed after providing typed descriptions of *all* nine thoughts.

As was done in Experiment 1, if a participant did not provide a response to a thought probe, this was coded as a failure and the corresponding ratings were removed from all analyses. Additionally, if a participant provided the same response to more than one thought prompt, only the first response was kept, and the rest were coded as failures and removed from the analyses. The failure rates for each thought type were: 4.35% (unexpected), 3.48% (IAM), and 5.38% (ruminative). In total, 3622 out of 3789 thoughts/memories were recalled (167 failures).

#### Materials

Materials are the same as those used in Experiment 1 with the addition of the AMCQ (Boyacioglu & Akfirat, 2015) and the inclusion of separate scales for positive and negative affect. In addition, we used the term ‘repetitive thought’ rather than ‘repetitive episode’ for the rumination appraisal items, the term ‘repetitive thought’ rather than ‘a series of repetitive thoughts’ for the rumination recall prompt, and the term ‘unexpected thought’ rather than ‘a thought that came out of the blue’ for the unexpected thought recall prompt (Table 1). Additionally, the word ‘unexpected’ was used rather than ‘spontaneous’ for the spontaneity appraisal dimension. For all appraisal items (including those from the AMCQ), wording was changed to reflect the type of thought being probed: event/memory (IAM), repetitive thought, or unexpected thought. Additionally, all questions were changed to 1–7 scales to provide consistency (except the dimension of mental image vividness). All replication dimensions and corresponding statements can be viewed in Table 2, while the dimensions and corresponding statements for the AMCQ can be viewed in Online Resource 1.

#### Procedure

Upon granting informed consent, participants were prompted to recall and describe three instances each of IAM, unexpected thought, and ruminative thought. After providing typed descriptions of *all* thoughts, participants answered a series of questions pertaining to the appraisal of each thought recalled. Lastly, participants completed a demographics questionnaire and were thoroughly debriefed on the purpose of the experiment.

### Results

Due to the repeated measures nature of our data, all analyses were run using linear mixed effects regression (or logistic mixed effects regression for identifiable cue) analyses using the *lme4* package in R using the same method as in Experiment 1 (Bates et al., 2015). For the AMCQ, each dimension was first scored by averaging the items in each dimension by a participant.

As mentioned above, we set the *α* to 0.001 (equivalent to a Bonferroni correction) to minimize egregious Type 1 errors, which is important given that we assessed the differences of 36 content variables in this follow-up experiment. Below, we present the results for all appraisal dimensions (minus the AMCQ), rather than only the dimensions for which there was an a priori prediction (as in Experiment 1 results), as achieving adequate power was ensured for this replication and extension study.

We first present the results regarding our a priori predictions, regardless of replication (i.e., dimensions regarding identifiable cues, surprising in content, surprising in timing, and unexpected). We then present the results for the exploratory dimensions that replicated across experiments, followed by the results that partially replicated across experiments. In this case, the main effects were replicated, but they did not have identical patterns for the pairwise comparisons. We are primarily focused on the comparisons with unexpected thought (unexpected thought vs. IAM; unexpected thought vs. ruminative thought), but we nevertheless report and discuss all comparisons for completeness. This section, therefore, includes findings that were replicated in terms of comparisons with unexpected thought, even if the other comparisons were not consistent. Finally, we present the results that did not replicate across experiments.

Once again, most of our numeric results can be found in the tables: means, standard deviations, and Cohen’s *d* values are provided in Table 6; Chi-square values, *b* coefficients, and *p*-values are provided in Table 7.

**Table 6:** Experiment 2 descriptive statistics and effect sizes

Appraisal dimension	Unexpected M (SD)	IAM M (SD)	Ruminative M (SD)	Cohen's *d* unexpected-IAM	Cohen's *d* unexpected-ruminative	Cohen's *d* IAM-ruminative
A priori dimensions						
Cue (proportion)	0.55 (0.37)	0.67 (0.34)	0.64 (0.34)	− 0.34*	− 0.26*	0.08
Surprising in content	4.32 (1.56)	3.73 (1.51)	3.34 (1.48)	0.38*	0.65*	0.27*
Surprising in timing	4.17 (1.45)	3.83 (1.47)	3.43 (1.48)	0.23*	0.50*	0.27*
Unexpected	4.66 (1.21)	4.15 (1.30)	3.72 (1.39)	0.41*	0.72*	0.32*
Replicated exploratory dimensions						
New info	3.47 (1.49)	3.00 (1.48)	3.07 (1.38)	0.32*	0.28*	− 0.05
Involuntary	4.41 (1.42)	4.09 (1.41)	3.75 (1.43)	0.22*	0.46*	0.24*
Task-unrelated	4.02 (1.36)	3.77 (1.48)	3.64 (1.36)	0.18*	0.28*	0.09
Goal	3.02 (1.43)	2.84 (1.51)	3.38 (1.42)	0.12	− 0.25*	− 0.36*
Emotional intensity	3.78 (1.27)	4.12 (1.15)	4.14 (1.26)	− 0.28*	− 0.29*	− 0.02
Partially-replicated exploratory dimensions						
Thought-unrelated	4.23 (1.35)	4.10 (1.42)	3.83 (1.38)	0.10	0.29*	0.19*
Personally immoral	3.44 (1.49)	2.78 (1.48)	2.99 (1.40)	0.44*	0.31*	− 0.15
Socially immoral	3.35 (1.53)	2.73 (1.47)	2.92 (1.43)	0.41*	0.29*	− 0.13
Beneficial in time	3.21 (1.41)	3.51 (1.39)	3.09 (1.32)	− 0.21*	0.09	0.31*
Beneficial now	3.25 (1.41)	3.62 (1.34)	3.22 (1.36)	− 0.27*	0.02	0.30*
Solve problem	3.07 (1.42)	2.78 (1.48)	3.24 (1.41)	0.20*	− 0.12	− 0.31*
Self-relevance	3.39 (1.41)	3.52 (1.47)	3.81 (1.41)	− 0.09	− 0.30*	− 0.21*
Positive emotion	3.45 (1.42)	4.23 (1.56)	3.18 (1.40)	− 0.53*	0.19*	0.71*
Negative emotion	4.01 (1.52)	3.50 (1.54)	4.37 (1.47)	0.33*	− 0.25*	− 0.58*
Mental image vividness	2.41 (0.97)	2.76 (0.73)	2.50 (0.98)	− 0.41*	− 0.09	0.30*
Non-replicated exploratory dimensions						
Value	3.52 (1.43)	4.02 (1.39)	3.93 (1.35)	− 0.35*	− 0.30*	0.06
Insight to others	2.98 (1.43)	3.23 (1.51)	3.00 (1.45)	− 0.17*	− 0.01	0.16*
Insight to self	3.27 (1.42)	3.16 (1.46)	3.35 (1.41)	0.08	− 0.05	− 0.13

*p < 0.001

**Table 7:** Experiment 2 Chi-squares, *b* coefficients, and *p* values

Appraisal dimension	Main effect (*Χ*^*2*^) (*p* value) *df* = 2	Pairwise comparisons (*b*) (*p* value)			
		Unexpected-IAM	Unexpected-ruminative	IAM-ruminative	Pattern^a^
A priori dimensions					
Cue (proportion)	51.42 (*p* < 0.001)*	− 0.63 (*p* < 0.001)*	− 0.50 (*p* < 0.001)*	0.13 (*p* = 0.329)	UT < RUM = IAM
Surprising in content	197.87 (*p* < 0.001)*	0.60 (*p* < 0.001)*	1.01 (*p* < 0.001)*	0.41 (*p* < 0.001)*	RUM < IAM < UT
Surprising in timing	111.73 (*p* < 0.001)*	0.35 (*p* < 0.001)*	0.76 (*p* < 0.001)*	0.42 (*p* < 0.001)*	RUM < IAM < UT
Unexpected	209.50 (*p* < 0.001)*	0.51 (*p* < 0.001)*	0.95 (*p* < 0.001)*	0.45 (*p* < 0.001)*	RUM < IAM < UT
Replicated exploratory dimensions					
New info	63.13 (*p* < 0.001)*	0.48 (*p* < 0.001)*	0.40 (*p* < 0.001)*	− 0.08 (*p* = 0.479)	IAM = RUM < UT
Involuntary	111.60 *p* < 0.001)*	0.34 (*p* < 0.001)*	0.67 (*p* < 0.001)*	0.33 (*p* < 0.001)*	RUM < IAM < UT
Task-unrelated	30.48 (*p* < 0.001)*	0.27 (*p* < 0.001)*	0.38 (*p* < 0.001)*	0.12 (*p* = 0.242)	RUM = IAM < UT
Goal	72.60 (*p* < 0.001)*	0.17 (*p* = 0.023)	− 0.38 (*p* < 0.001)*	− 0.55 (*p* < 0.001)*	IAM = UT < RUM
Emotional intensity	47.24 (*p* < 0.001)*	− 0.35 (*p* < 0.001)*	− 0.36 (*p* < 0.001)*	− 0.01 (*p* = 0.988)	UT < IAM = RUM
Partially-replicated exploratory dimensions					
Thought-unrelated	35.60 (*p* < 0.001)*	0.13 (*p* = 0.132)	0.40 (*p* < 0.001)*	0.27 (*p* < 0.001)*	RUM < IAM = UT
Personally immoral	115.66 (*p* < 0.001)*	0.66 (*p* < 0.001)*	0.45 (*p* < 0.001)*	− 0.21 (*p* = 0.002)	IAM = RUM < UT
Socially immoral	119.29 (*p* < 0.001)*	0.63 (*p* < 0.001)*	0.46 (*p* < 0.001)*	− 0.17 (*p* = 0.016)	IAM = RUM < UT
Beneficial in time	43.78 (*p* < 0.001)*	− 0.30 (*p* < 0.001)*	0.11 (*p* = 0.226)	0.41 (*p* < 0.001)*	RUM = UT < IAM
Beneficial now	48.98 (*p* < 0.001)*	− 0.37 (*p* < 0.001)*	0.03 (*p* = 0.908)	0.40 (*p* < 0.001)*	RUM = UT < IAM
Solve problem	52.91 (*p* < 0.001)*	0.28 (*p* < 0.001)*	− 0.16 (*p* = 0.028)	− 0.44 (*p* < 0.001)*	IAM < UT = RUM
Self-relevance	44.78 (*p* < 0.001)*	− 0.13 (*p* = 0.141)	− 0.44 (*p* < 0.001)*	− 0.31 (*p* < 0.001)*	UT = IAM < RUM
Positive emotion	225.83 (*p* < 0.001)*	− 0.79 (*p* < 0.001)*	0.25 (*p* = 0.001)*	1.04 (*p* < 0.001)*	RUM < UT < IAM
Negative emotion	140.02 (*p* < 0.001)*	0.50 (*p* < 0.001)*	− 0.37 (*p* < 0.001)*	− 0.88 (*p* < 0.001)*	IAM < UT < RUM
Mental image vividness	81.87 (*p* < 0.001)*	− 0.35 (*p* < 0.001)*	− 0.10 (*p* = 0.043)	0.25 (*p* < 0.001)*	UT = RUM < IAM
Non-replicated exploratory dimensions					
Value	60.75 (*p* < 0.001)*	− 0.51 (*p* < 0.001)*	− 0.42 (*p* < 0.001)*	0.09 (*p* = 0.413)	UT < RUM = IAM
Insight to others	21.32 (*p* < 0.001)*	− 0.25 (*p* < 0.001)*	− 0.01 (*p* = 0.994)	0.24 (*p* < 0.001)*	UT = RUM < IAM
Insight to self	10.41 (*p* = 0.005)	NA	NA	NA	NA

*p < 0.001

^a^*UT* = unexpected thought; *RUM* = ruminative thought

#### Number of cues

Pairwise comparisons indicated that unexpected thought had significantly fewer cues than both IAM and ruminative thought. There was no significant difference between IAM and ruminative thought.

#### Surprising in content

Pairwise comparisons indicated that unexpected thought had significantly more surprising content than both IAM and ruminative thought. Further, IAM was significantly more surprising in content compared to ruminative thought.

#### Surprising in timing

Pairwise comparisons indicated that unexpected thought was significantly more surprising in timing compared to IAM and ruminative thought. Further, IAM was significantly more surprising in timing compared to ruminative thought.

#### Unexpected

Pairwise comparisons indicated that unexpected thought felt significantly more unexpected than IAM and ruminative thought. Additionally, IAM felt significantly more unexpected than ruminative thought.

#### Exploratory dimensions: replicated results

Table 7 provides the results regarding the exploratory dimensions which replicated across experiments: new information, involuntary, task-unrelated, goal, and emotional intensity.

In general, these patterns suggest that unexpected thought is less likely to have identifiable cues compared to IAM and ruminative thought, while also being the most likely to provide new information, be surprising in timing, unexpected, task-unrelated, and involuntary. In contrast, IAM is the most likely to produce vivid mental images, while ruminative thought is the most likely to evoke negative emotion and is the least surprising in timing.

#### Thought-unrelated

Unexpected thought was significantly more thought-unrelated than ruminative thought. IAM was also significantly more thought-unrelated than ruminative thought. There was no significant difference between unexpected thought and IAM.

#### Personally immoral

Unexpected thought was reported to contradict personal morals significantly more than IAM and ruminative thought. There was no significant difference between IAM and ruminative thought.

#### Socially immoral

Unexpected thought was reported to contradict social morals significantly more than IAM and ruminative thought. There was no significant difference between IAM and ruminative thought.

#### Beneficial in time

Unexpected thought was significantly less beneficial than IAM. There was no significant difference between unexpected thought and ruminative thought. Finally, IAM was significantly more beneficial in time compared to ruminative thought.

#### Beneficial now

Unexpected thought was significantly less beneficial now compared to IAM. There was no significant difference between unexpected thought and ruminative thought. However, IAM was significantly more beneficial than ruminative thought.

#### Solve problem

Unexpected thought was significantly more helpful in solving a problem than IAM. Further, ruminative thought was significantly more helpful in solving a problem than IAM. There was no significant difference between unexpected thought and ruminative thought.

#### Self-relevance

Ruminative thought was rated as significantly more self-relevant compared to unexpected thought and IAM. There was no significant difference between unexpected thought and IAM.

#### Positive emotion

IAM was significantly more positive in emotion compared to unexpected thought and ruminative thought. Further, unexpected thought was significantly more positive in emotion compared to ruminative thought.

#### Negative emotion

Ruminative thought was significantly more negative in emotion compared to IAM and unexpected thought. Unexpected thought was also significantly more negative in emotion compared to IAM.

#### Mental image vividness

IAM was significantly more vivid compared to unexpected thought and ruminative thought. There was no significant difference between unexpected thought and ruminative thought.

#### Exploratory dimensions: non-replicated results

Table 7 provides the results regarding the exploratory dimensions which did not replicate across experiments; these include value, others-insight, and self-insight.

### Discussion

Our central aim in conducting Experiment 2 was to replicate the findings from our pilot Experiment 1 while addressing possible limitations in our methods. Participants were thus instructed to appraise their thoughts after first providing typed descriptions of all nine thoughts. As in Experiment 1, we found that unexpected thought had significantly fewer cues, was more surprising in both content and timing, and felt significantly more task-unrelated and unexpected (or spontaneous in Experiment 1) compared to IAM and ruminative thought. We also replicated the findings that unexpected thought was significantly more likely to be perceived as providing new information and be less emotionally intense compared to IAM and ruminative thought.

It is worth noting that a number of findings only partially replicated across experiments—that is, they replicated with respect to the main effects but did not have identical patterns for the pairwise comparisons. We put more stock in the findings from Experiment 2, given that it was well-powered, and we ultimately believe it is worthwhile to focus on the dimensions that were consistent across both experiments (see General Discussion).

## Can we predict the type of involuntary thought using machine learning?

As a final research question, we were interested in whether we could accurately classify the type of thought recalled using only the ratings from the appraisal dimensions using machine learning. Given that all three forms of thought represent an involuntary phenomenon, this question was designed to shed light on how “separable” the three forms of involuntary thoughts are based on appraisals alone, while also giving more information on which types of thoughts may be misclassified for one another. For this analysis, we used all of the dimensions from Experiment 2 and did not include any Experiment 1 data. However, we removed the “unexpected” dimension so as to not bias the models. We chose to use Experiment 2 data due to differences in power between the studies.

We used *K*-fold cross-validation, such that all thought instances were first divided into K (in our case, 5) random subsets. The algorithm is then trained on *K-1* groups to “learn” the patterns of appraisals in the data and then make predictions on a “held out” subset (i.e., the held out “test set”); this process is repeated until all subsets have been held out as the test set. We completed these analyses to help us understand whether the appraisal patterns are generalizable and predictive—that is, can you take a set of appraisal patterns and make a reasonably accurate prediction with no other information about the person’s thought?

In total, three common classification algorithms (Random Forest, SVM, and Naïve Bayes) were applied to the dataset, and accuracies and kappa values for all models can be viewed in Table 8. Accuracy is defined as the number of correct predictions out of all data points, ranging from 0 to 100%. Kappa is defined as the degree of agreement between the true data points and the predicted data points, ranging from − 1 to 1, and can be converted to represent how well a model is performing above chance.

**Table 8:** Classification algorithms, accuracy, and kappa

Classifier	Average accuracy	Kappa
SVM	0.498	0.247
Random forest	0.499	0.248
Naïve bayes	0.479	0.219

Baseline (or chance) accuracy for our models would stand at 33%, and the Random Forest classifier performed the best amongst all models, achieving an accuracy of roughly 50%. Our model performed 25% above chance levels (kappa = 0.25), suggesting that although the three forms of involuntary thought may not be completely dissociable, the appraisal dimensions provide a strong enough signal to make relatively accurate classifications. Table 9 contains the confusion matrix for the Random Forest model. One can also observe from this matrix that unexpected thought and ruminative thought were more likely to be “confused” with one another, while IAM was more easily classified. This suggests that unexpected and ruminative thoughts are more closely related to one another than to IAMs, possibly because both experiences rely less so on one’s past memories.

**Table 9:** Confusion matrix for best model (random forest)

Predicted	Actual		
	IAM	Ruminative	Unexpected
IAM	610	235	252
Ruminative	304	581	341
Unexpected	305	379	615

In general, the fact that unexpected thought, IAM, and ruminative thought are not classified with even higher accuracy is telling; it may speak to a general set of overlapping features between the three types of involuntary thought, with some appraisals (e.g., cues) being more likely to separate paradigmatic cases of such thoughts.

The following set of qualitative analyses are purely exploratory, but we include them to provide more insight into the data. After observing that the machine learning model did not equally “confuse” the misclassified thoughts, we conducted an exploratory qualitative content analysis to better understand the source of confusion and to identify any distinguishing markers that may not be captured by the appraisal approach. To do this, we extracted 100 randomly chosen unexpected thought recalls and the first author qualitatively assessed if any post-hoc clusters were identifiable—that is, was there anything about the thoughts that seemed to stick out or group them together? We gained three key qualitative insights.

First, some of the unexpected thoughts contained episodic content (7%) while others were triggered by cues in the environment—possibly blurring the lines between the three types of involuntary thinking. Second, despite this blurring, one identifiable characteristic of the unexpected thoughts reported was the “What if I did [insert some low probability event]” nature of many of the thoughts. This included negative simulative scenarios surrounding socially-deviant acts (29%) and positive simulative scenarios (6%). The negative simulations observed here are akin to past work on what is known as the “high place phenomenon” in which one feels a sudden urge to jump when in a high place, and such experiences appear to be common amongst the general population (Hames et al., 2012; Teismann et al., 2020). Table 10 provides examples of the negative simulations reported. Finally, many of the thoughts also contained new information or provided insight (26%; examples of these are also included in Table 10). This latter finding is in line with the results of both Experiment 1 and 2 in which unexpected thoughts were significantly more likely to provide new information compared to both IAM and ruminative thought.

**Table 10:** Categories of unexpected thought

Negative simulation	1. When driving my car, I thought of what would happen if my car randomly broke down in that moment and what I would do. 2. Once I was at my job (daycare) and I was thinking about what would happen if there was a tornado. 3. I was driving and thought what if I just drove off the side of the road.
Insight/new information	1. I randomly thought that everything must happen for a reason so there is no need to worry. I was in a bad place at the time. I basically came to the conclusion that our environments and our genetics determine who we are, and nobody chooses the circumstance they are born into or the genes they have to live out that circumstance. This took the burden off me and allowed me to relax. 2. When I was taking my most recent biology exam I had not studied nearly enough and I could not remember most of the content. When I was looking at a math problem that required an equation, suddenly it popped into my head and I did not even remember learning it in the first place. 3. I think when I was thinking about maybe learning how to surf. This is not something I would normally do.

## General discussion

The current set of studies assessed the appraisal dimensions of three common forms of involuntary thinking (unexpected thoughts, involuntary autobiographical memories, and ruminative thoughts) to determine if these thoughts have distinct appraisal patterns. We were particularly focused on how unexpected thought may differ from IAM and rumination, two widely studied forms of involuntary thinking. Although previous theories have proposed that the phenomenological experience of surprise or unexpectedness may arise due to fewer identifiable cues and the extent of surprising content (Mills et al., 2021), there is no empirical work to support this claim. Further, given the overlap with other involuntary thought processes, it is not clear if unexpected thoughts are phenomenologically distinct from IAM and rumination, or along which dimensions they may be separable. We based our methods on the emotion appraisal literature (Ellsworth & Scherer, 2003; Smith & Ellsworth, 1985) and Andrews-Hanna et al. (2013) by conducting two experiments in which participants appraised three instances each of unexpected thought, IAM, and ruminative thought.

### Main findings

Across two experiments, we found that unexpected thoughts were dissociable from IAM and ruminative thought on a number of appraisal dimensions; Table 11 indicates the appraisal dimensions that were specific to each type of thought in both experiments. For example, unexpected thoughts were characterized as having the fewest cues, being the most surprising in timing and content, and providing the greatest amount of new information compared to IAM and ruminative thought. Unexpected thoughts also felt the most spontaneous, involuntary, and task-unrelated. IAM, in contrast, was characterized as the most vivid. Finally, ruminative thought was the least surprising in timing, the least spontaneous, and the most affectively negative.

**Table 11:** Dimensions specific to each thought type across experiments

Unexpected	Ruminative	IAM
Cue (−)	Surprising in timing (−)	Mental image vividness (+)
Surprising content (+)	Spontaneous/unexpected (−)	
Surprising timing (+)	Involuntary (−)	
Spontaneous/unexpected (+)	Thought-unrelated (−)	
New info (+)	Goal (+)	
Involuntary (+)	Positive emotion (−)	
Emotional Intensity (−)	Negative emotion (+)	
Task-unrelated (+)		

This pattern of results largely supports previous theories on the phenomenological experience of unexpected thoughts—particularly in that they have less identifiable causes compared to other forms of spontaneous thinking. These appraisals also support prior work on IAM and ruminative thought (Barzykowski & Niedźwieńska, 2016; Berntsen & Hall, 2004; Nolen-Hoeksema, 2000), such that IAM is typically vivid and rumination is more likely to be affectively negative and the least surprising. Aside from these delineations, one of the particularly interesting findings is that unexpected thoughts were appraised as providing more new information than IAM and ruminative thoughts. This is in line with theoretical ideas from prior work suggesting that there is likely some benefit for such unexpected thoughts (Mills et al., 2021); they provide us with novelty in our thought streams so we can learn and simulate new things, even unintentionally. This is also in line with previous work on sudden insights, where ideas come to us out of the blue (Laukkonen et al., 2018; Topolinski & Reber, 2010). However, not all unexpected thoughts were found to contain insightful information, suggesting that unexpected thoughts are dissociable from moments of insight. Nevertheless, they may sometimes offer new information for us to work with. Additionally, the finding that unexpected thoughts were most likely to contain insightful information also aligns with previous work suggesting that task-unrelated thought may promote creative incubation (Baird et al., 2012; though see Murray et al., 2021). However, as mentioned above, task-unrelated thoughts are unlikely to feel unexpected in nature by default (Baird et al., 2011; Seli et al., 2016).

### Implications for theory and future research

There are varying conceptualizations of spontaneous thought within the literature, and we think this work may help situate different forms of involuntary thought in that space. Perhaps the best approach is to conceive of IAM, ruminative thought, and unexpected thought as separate forms of spontaneous thought based on their differential appraisals. In this way, spontaneous thought may be used as an umbrella term which also includes dreaming and mind wandering (Berntsen, 2021; Christoff et al., 2016). Here, however, we provide empirical evidence for possible ways that, under this umbrella of spontaneity, we can separate conceptually and experientially distinct forms of spontaneous thinking. We specifically focus on the experience of unexpectedness in thought which may reflect paradigmatic cases of spontaneous thought in that it has no easily attributable causes—such thoughts are unbidden and unexpected based on timing and content. Consistent with our findings, various forms of involuntary thoughts may *feel* more or less spontaneous based on these appraisal dimensions.

Our findings support Mills et al. (2021) conceptualization of wayward transitions, referred to as unexpected thoughts here, as invoking the feeling of surprise due to their low occurrence probability. Namely, unexpected thoughts were appraised as being more surprising in terms of their content and timing than either IAM or ruminative thoughts. Unexpected thoughts appear to be more independent of goals, affect, and/or other internal and external stimuli than other spontaneous thoughts (i.e., their causes are less obvious).

Unexpected thoughts were appraised as also providing more new information compared to IAM and ruminative thoughts. This may, in part, simply reflect that the contents of unexpected thoughts are less common and, in this sense, more novel. However, it is also possible that their manner of arising influences their perceived spontaneity regardless of whether their contents are truly novel. We provide some support for this idea, showing that thoughts arising through automatic constraints (e.g., affectively salient information, environmental cues, etc.) engender dampened appraisals of thought spontaneity (Christoff et al., 2016). Notably, Mandler (2007) proposed that unexpectedness may be a dimension that underlies IAMs specifically, as some involuntary memories may be more or less surprising depending on their relevance to the current situation. This also supports the idea that there may be a graded membership of spontaneous thoughts due to differing levels of surprise in content.

So, how should we view these different forms of spontaneous thought as we continue to make scientific progress in this field? First, past literature suggests that involuntary, or spontaneous, thoughts are ubiquitous in various forms and that understanding them is an important step in modeling human cognition (Berntsen, 2010; Christoff et al., 2004, 2016; Fox et al., 2015). However, unexpected thoughts have been studied on their own less often. Although we found that unexpected thoughts were the most task-unrelated, unexpected thoughts are different from the traditional view of mind wandering, where people are not necessarily surprised by the content of their thought and often engage in deliberate planning when they go off-task (Christoff et al., 2016; Mooneyham & Schooler, 2013). Second, despite some evidence of dissociation among the forms of spontaneous thought, we argue that our results do not speak to a complete delineation between these forms of involuntary thinking. Instead, we identify some specific dimensions that appear to separate them. We believe our results, similar to the approaches taken by others (Andrews-Hanna et al., 2013; Moors et al., 2013), speak to a dimensional approach to categorizing spontaneous thoughts based on how they arise (or how they are perceived to arise). For example, an IAM may *feel* more spontaneous if a relevant cue is not available. Similarly, unexpected thoughts may involve autobiographical content, and there may be individual differences with respect to how people attribute the causes of their own thoughts.

It should also be mentioned that other types of involuntary thought have been discussed in different literatures, such as involuntary prospective memory and intrusive thoughts (Jones & Fernyhough, 2009; McDaniel & Einstein, 2000). While a discussion of other types of involuntary thought is beyond the scope of this paper, future research may investigate the relationship between unexpected thoughts and other forms of involuntary thoughts. Additionally, future work may also investigate the phenomenology of involuntary thoughts via virtual reality (VR) paradigms. VR is emerging as an advantageous methodological tool due to its immersive nature, with previous research suggesting that VR may assist in practicing mindfulness (Seabrook et al., 2020) as well as treating conditions such as posttraumatic stress disorder (PTSD) (Gonçalves et al., 2012; Rothbaum et al., 1999). Notably, VR has also been used to induce feelings of déjà vu (Cleary et al., 2012), which may also be a form of involuntary thinking due to its highly constrained nature (i.e., cues in the environment must be closely aligned to a previous experience to induce feelings of déjà vu; Barzykowski & Moulin, 2022). Future work may thus entail inducing forms of spontaneous thoughts via VR paradigms to examine their phenomenology in real-time.

While the appraisal method has been foundational in research on emotion (e.g., Smith & Ellsworth, 1985), we applied it in a novel way in the current studies, combined with procedures for data collection and coding from other literatures. Some limitations of our methods should be acknowledged. Participants were instructed to recall previously experienced thoughts, and this may be a difficult task for a few reasons. For example, participants may not feel comfortable reporting their thoughts due to the emotional impact of reliving such experiences, as some thoughts and/or memories may be particularly emotionally valenced or intense. Further, participants may not feel comfortable if they believe their thoughts/memories do not align with their personal or societal morals. Finally, participants may simply have trouble recollecting such experiences, particularly unexpected thoughts. As such, well-documented inaccuracies in self-reports (e.g., Schwarz, 1999) may have emerged in the current paradigm, such that some participants may have generated responses to the prompts that were not true experiences of their own. Future work could employ alternative methods to evaluate involuntary thoughts, such as experience sampling or methods in which participants appraise a range of sample thoughts rather than recall their own. Future work might also incorporate linguistic analyses to determine whether there are features of the text-based recalls (e.g., commonly used words, affective language) that can be extracted to further delineate the three types of thought that were our focus.

Taken together, the results of the two studies presented here represent a fruitful first pass at distinguishing the characteristics of unexpected thoughts from IAMs and ruminative thoughts. These results also support previous theories that spontaneous thinking manifests in various forms. We believe this work contributes to a more nuanced understanding of involuntary thought processes and what makes us feel spontaneity in our thoughts.

## Supplementary Information

Below is the link to the electronic supplementary material.

Supplementary file1 (PDF 175 KB)

## Data Availability

All of our numeric data are available on OSF (https://osf.io/wdgcy/?view_only=3161fc311aaa4ad2891327a9d88bcf3c); full recall data are also available upon request.

## Footnotes

1 These thoughts may never truly be spontaneous in the sense that they do not literally come from nowhere (neurally or otherwise); however, they tend to share a lack of volition in terms of how the mental state arises and thus often lack a sense of agentic causality.

2 We acknowledge the terminology debate within the literature and thus distinguish unexpected thought from other types of thought such as mind wandering and self-generated thought.

3 Given the imbalance of conditions, we reran all results to include counterbalance as a fixed effect, and the results by Thought Condition were not changed.
