# Supplementary material for: Investigating the appraisal structure of spontaneous thoughts: evidence for differences among unexpected thought, involuntary autobiographical memories, and ruminative thought
Source: Psychol Res. 2023 Mar 31;87(8):2345–64. doi: 10.1007/s00426-023-01814-y (PMC10497430; doi:10.1007/s00426-023-01814-y)
Supplement: Supplementary file 1 — Supplementary file1 (PDF 175 KB) [file 426_2023_1814_MOESM1_ESM.pdf]

Investigating the Appraisal Structure of Spontaneous Thoughts: Evidence for Differences Among Unexpected Thought, Involuntary Autobiographical Memories, and Ruminative Thought

Cati Poulos[1,2], Andre Zamani[3], David Pillemer[1], Michelle Leichtman[1], Kalina Christoff[3,4], Caitlin Mills[1,2]

[1]Department of Psychology, University of New Hampshire, Durham, NH, USA

[2]Department of Educational Psychology, University of Minnesota Twin Cities, Minneapolis, MN, USA

[3]Department of Psychology, and [4]Centre for Brain Health, University of British Columbia, Vancouver, British Columbia, Canada

Corresponding Author (Current Address)

Cati Poulos

56 E River Road

University of Minnesota Twin Cities

Minneapolis, MN 55455

mcatipoulos@gmail.com

Table 1

AMCQ: Appraisal Dimensions, Scales, and Appraisal Items (Adapted from Boyacioglu & Akfirat, 2015)

**Appraisal Dimension**	**Question**<br>**Scale: 1 (Completely disagree) to 7 (Completely agree)**
Vividness	(1) I remember the **unexpected thought/repetitive thought/memory** vividly<br>(2) I remember the **unexpected thought/repetitive thought/memory** very clearly<br>(3) The things I experienced during the **unexpected thought/repetitive thought/memory** are so vivid that I feel as though it just happened yesterday<br>(4) I can remember the situation in which the **unexpected thought/repetitive thought/memory** occurred vividly, as though I were there<br>(5) If I asked, I could make a film about the **unexpected thought/repetitive thought/memory** that would depict exactly what happened, because I remember it so clearly
Belief in accuracy	(1) I think that the **unexpected thought/repetitive thought/memory** may have occurred differently than how I remember it (R)<br>(2) I am seriously doubtful that the **unexpected thought/repetitive thought/memory** occurred in the way I remember it (R)<br>(3) The **unexpected thought/repetitive thought/memory** occurred exactly the way I remember it<br>(4) As I remember the **unexpected thought/repetitive thought/memory**, I have not imagined or fabricated anything that did not occur<br>(5) I am sure that the **unexpected thought/repetitive thought/memory** occurred in the way I remember it
Place details	(1) I can remember the city in which the **unexpected thought/repetitive thought/memory** took place<br>(2) I can remember the country in which the **unexpected thought/repetitive thought/memory** took place
Sensory details	(1) I can remember my body position or my posture or how I acted during the **unexpected thought/repetitive thought/memory**<br>(2) As I remember this **unexpected thought/repetitive thought/memory**, I can see the details or the colors of the things related to the **unexpected thought/repetitive thought/memory** (such as the furniture or the wallpaper)<br>(3) I can remember tactile details (such as temperature or pain) about the **unexpected thought/repetitive thought/memory**.<br>(4) As I remember this **unexpected thought/repetitive thought/memory**, I can sense the odor (such as damp or perfume of someone close to you) surrounding the **unexpected thought/repetitive thought/memory**<br>(5) As I remember this **unexpected thought/repetitive thought/memory**, I can hear the sounds that I heard during the **unexpected thought/repetitive thought/memory** in my mind
Accessibility	(1) I had to search my memory to recall this **unexpected thought/repetitive thought/memory** (R)<br>(2) I had to think for a while to recall this **unexpected thought/repetitive thought/memory** (R)<br>(3) I do not recall this **unexpected thought/repetitive thought/memory** very often (R)
Sharing	(1) I often share this **unexpected thought/repetitive thought/memory** with others<br>(2) I often talk about this **unexpected thought/repetitive thought/memory** with my friends or family

	(3) Since it happened, I have talked about this **unexpected thought/repetitive thought/memory** with others many times<br>(4) I have not talked about the **unexpected thought/repetitive thought/memory** with others (R)<br>(5) I do not share this **unexpected thought/repetitive thought/memory** with others (R)
Observer perspective	(1) As I remember the **unexpected thought/repetitive thought/memory**, I feel as though I am seeing the **event** as an outside observer<br>(2) As I remember the **unexpected thought/repetitive thought/memory**, I feel as though I am watching a short film about someone else<br>(3) As I remember the **unexpected thought/repetitive thought/memory**, I feel as though I am looking at the past me and others around me from above or far away
Field perspective	(1) As I remember the **unexpected thought/repetitive thought/memory**, I imagine it again through my own eyes<br>(2) I view this **unexpected thought/repetitive thought/memory** through my own eyes, from my own perspective<br>(3) As I remember the **unexpected thought/repetitive thought/memory**, I feel as though I am seeing the **unexpected thought/repetitive thought/memory** out of my own eyes rather than as an outside observer
Narrative coherence	(1) This **unexpected thought/repetitive thought/memory** comes back to me in disjointed pieces (R)<br>(2) This **unexpected thought/repetitive thought/memory** comes back to me in disjointed flashbacks (R)<br>(3) As I remember the **unexpected thought/repetitive thought/memory**, there are gaps and some things I cannot remember in the storyline (R)<br>(4) The order of events in the **unexpected thought/repetitive thought/memory** is incoherent and confusing (R)<br>(5) I remember the **unexpected thought/repetitive thought/memory** in chronological order (before, during and after)
Recollection	(1) As I remember the **unexpected thought/repetitive thought/memory**, I feel as though I am travelling to the time it happened<br>(2) As I remember the **unexpected thought/repetitive thought/memory**, I feel as though I travelled back and become the same person in the **unexpected thought/repetitive thought/memory**<br>(3) As I remember the **unexpected thought/repetitive thought/memory**, I feel as though I am reliving it<br>(4) As I remember the **unexpected thought/repetitive thought/memory**, I feel as if I am thinking the same things or feeling the same emotions as I did during the **unexpected thought/repetitive thought/memory**<br>(5) As I think about the **unexpected thought/repetitive thought/memory**, I actually remember it rather than just knowing that it happened
Emotional valence	(1) The experience I recall is quite negative (R)<br>(2) The experience I recall is quite positive<br>(3) My feelings at the time of the **unexpected thought/repetitive thought/memory** were quite negative (R)<br>(4) My overall feeling about the **unexpected thought/repetitive thought/memory** is quite negative (R)<br>(5) My feelings at the time of the **unexpected thought/repetitive thought/memory** were quite positive<br>(6) My overall feeling about the **unexpected thought/repetitive thought/memory** is quite positive
Emotional intensity	(1) While remembering the **unexpected thought/repetitive thought/memory** now, my feelings are quite weak (R)<br>(2) While remembering the **unexpected thought/repetitive thought/memory** now, my feelings are quite intense<br>(3) My memory of this **unexpected thought/repetitive thought/memory** evokes very weak emotions in me (R)<br>(4) When I focus the **unexpected thought/repetitive thought/memory**, I feel very intense emotions<br>(5) My memory of this **unexpected thought/repetitive thought/memory** evokes very strong emotions in me

Emotional distancing	(1) As I remember the **unexpected thought/repetitive thought/memory**, I bottle up my feelings<br>(2) While talking about this **unexpected thought/repetitive thought/memory**, I relate what happened rather than what I felt or thought.<br>(3) As I remember the **unexpected thought/repetitive thought/memory**, I smother my feelings<br>(4) As I remember the **unexpected thought/repetitive thought/memory**, although my feelings are important to me, I prefer to ignore them
Preoccupation with emotions	(1) This **unexpected thought/repetitive thought/memory** is still difficult to think about because of the feelings that the **unexpected thought/repetitive thought/memory** evokes<br>(2) As I remember the **unexpected thought/repetitive thought/memory**, my feelings are so intense that I cannot focus on any activity after that<br>(3) As I remember the **unexpected thought/repetitive thought/memory**, I feel lost in my thoughts because of my feelings.<br>(4) While talking about this **unexpected thought/repetitive thought/memory**, I feel such an intense need to share my feelings that I cannot tell the **unexpected thought/repetitive thought/memory** as a coherent story<br>(5) As I remember the **unexpected thought/repetitive thought/memory**, I cannot focus on anything except my feelings<br>(6) As I remember the **unexpected thought/repetitive thought/memory**, my feelings confuse me

Experiment 2 AMCQ Results

All dimensions and corresponding statements for the AMCQ are provided in Table 1. Additionally, means, standard deviations, and Cohen's *d* values are provided in Table 2, and Chi-square values, *b* coefficients, and *p*-values are provided in Table 3. For conciseness, we found a significant main effect of Condition for *all* appraisal dimensions with two exceptions (α = .001): field perspective (p = .349) and narrative coherence (p = .017). We thus refer readers to Table 3 for specific values regarding main effects, and below we focus on pairwise comparisons.

Vividness

Pairwise comparisons indicated that unexpected thought was significantly less vivid compared to IAM and ruminative thought. There was no significant difference between IAM and ruminative thought. These results are in line with the IAM literature, as memories that are easily able to be recalled are often the most vivid memories (Barzykowski & Niedźwieńska, 2016).

Accuracy

Unexpected thought recalls were reported to be accurate significantly less compared to IAM and ruminative thought. There was no significant difference between IAM and ruminative thought.

Place Details

Unexpected thought had significantly less place details compared to IAM. There was no significant difference between unexpected thought and ruminative thought. Further, IAM had significantly more place details than ruminative thought.

Sensory Details

Unexpected thought had significantly less sensory details compared to IAM. There was no significant difference between unexpected thought and ruminative thought. Further, IAM had significantly more sensory details than ruminative thought. Again, these results, along with the place details results, are in line with the IAM literature, as memories that are able to be recalled often contain the most detailed information (Barzykowski & Niedźwieńska, 2016).

Accessibility

Unexpected thought was significantly less accessible compared to both IAM and ruminative thought. Further, IAM was significantly less accessible compared to ruminative thought. These results may be explainable by the idea that unexpected thought and IAM are both cued events which may be seemingly unrelated to current internal or external states, making them less accessible to recall, while RUM is often characterized by a focus on personal concerns or goals.

Sharing

Unexpected thought was shared with others significantly less compared to IAM and ruminative thought. Further, IAM was shared with others significantly more compared to ruminative thought.

Observer Perspective

Unexpected thought was reported to have an observer perspective significantly less compared to IAM. There was no significant difference between unexpected thought and ruminative thought. Further, IAM was reported to have an observer perspective significantly more compared to ruminative thought.

Field Perspective

There was no significant main effect of Condition.

Narrative Coherence

No significant main effect of Condition was observed.

Recollection

Unexpected thought recalls were reported to induce a sense of reliving the original experience significantly less compared to IAM. Further, IAM recalls induced a sense of reliving the original experience significantly more compared to ruminative thought. There was no significant difference between unexpected thought and ruminative thought.

Valence

Unexpected thought was significantly less positive in valence compared to IAM. However, unexpected thought was significantly more positive in valence compared to ruminative thought. Further, IAM was significantly more positive in valence compared to ruminative thought.

Emotional Intensity

Unexpected thought was significantly less emotionally intense compared to IAM and ruminative thought. There was no significant difference between IAM and ruminative thought.

Emotional Distancing

IAM was significantly less likely to lead to a distancing from emotions compared to ruminative thought. There was no significant difference between unexpected thought and IAM or between unexpected thought and ruminative thought.

Preoccupation with Emotion

IAM was significantly less likely to lead to a preoccupation with emotions compared to ruminative thought. There was no significant difference between unexpected thought and IAM or between unexpected thought and ruminative thought.

Table 2

AMCQ Descriptive Statistics and Effect Sizes

Variable	UT<br>M (SD)	IAM<br>M (SD)	RUM<br>M (SD)	Cohen's d<br>UT-IAM	Cohen's d<br>UT-RUM	Cohen's d<br>IAM-RUM
Vividness	3.95 (1.26)	4.43 (1.20)	4.23 (1.30)	-0.39*	-0.22*	0.15
Accuracy	4.50 (1.00)	4.72 (1.00)	4.65 (1.00)	-0.23*	-0.15*	0.08
Place Details	5.28 (1.55)	5.70 (1.43)	5.39 (1.57)	-0.28*	-0.08	0.20*
Sensory Details	3.76 (1.26)	4.34 (1.24)	3.89 (1.34)	-0.46*	-0.11	0.34*
Accessibility	4.16 (1.18)	4.43 (1.20)	4.69 (1.24)	-0.22*	-0.44*	-0.21*
Sharing	3.34 (1.11)	3.82 (1.01)	3.52 (1.07)	-0.45*	-0.16*	0.28*
Observer Perspective	3.02 (1.43)	3.30 (1.48)	3.02 (1.45)	-0.20*	-0.01	0.19*
Field Perspective	4.55 (1.48)	4.63 (1.45)	4.59 (1.42)	-0.06	-0.03	0.03
Narrative Coherence	4.58 (1.05)	4.66 (1.10)	4.69 (1.00)	-0.08	-0.11	-0.02
Recollection	3.72 (1.22)	4.21 (1.24)	3.87 (1.25)	-0.40*	-0.13	0.27*
Valence	3.78 (1.19)	4.41 (1.28)	3.45 (1.18)	-0.51*	0.28*	0.78*
Emotional intensity	3.67 (0.94)	3.99 (0.85)	3.94 (0.91)	-0.35*	-0.29*	0.05
Distancing	3.07 (1.30)	2.97 (1.31)	3.24 (1.37)	0.08	-0.13	-0.20*
Preoccupation with Emotion	2.86 (1.36)	2.75 (1.34)	3.01 (1.36)	0.08	-0.11	-0.19*

*$p \leq .001$

Table 3

AMCQ Chi-Squares, b coefficients, and p values

Variable	Main Effect (X^2) (p value)	Pairwise Comparisons (b) (p value)		
		UT-IAM	UT-RUM	IAM-RUM
Vividness	77.20 (p < .001)*	-0.48 (p < .001)*	-0.30 (p < .001)*	0.19 (p = .002)
Accuracy	36.67 (p < .001)*	-0.23 (p < .001)*	-0.16 (p < .001)*	0.07 (p = .151)
Place Details	77.82 (p < .001)*	-0.42 (p < .001)*	-0.13 (p = .018)	0.29 (p < .001)*
Sensory Details	143.27 (p < .001)*	-0.58 (p < .001)*	-0.14 (p = .018)	0.44 (p < .001)*
Accessibility	87.77 (p < .001)*	-0.26 (p < .001)*	-0.52 (p < .001)*	-0.25 (p < .001)*
Sharing	72.51 (p < .001)*	-0.48 (p < .001)*	-0.20 (p = .001)*	0.27 (p < .001)*
Observer Perspective	45.96 (p < .001)*	-0.31 (p < .001)*	0.00 (p = .996)	0.31 (p < .001)*
Field Perspective	2.10 (p = .349)	NA	NA	NA
Narrative Coherence	8.11 (p = .017)	NA	NA	NA
Recollection	115.86 (p < .001)*	-0.51 (p < .001)*	-0.17 (p = .002)	0.35 (p < .001)*
Valence	267.05 (p < .001)*	-0.64 (p < .001)*	0.34 (p < .001)*	0.99 (p < .001)*
Emotional intensity	59.12 (p < .001)*	-0.33 (p < .001)*	-0.27 (p < .001)*	0.06 (p = .388)
Distancing	30.01 (p < .001)*	0.10 (p = .078)	-0.16 (p = .003)	-0.26 (p < .001)*
Preoccupation with Emotion	32.31 (p < .001)*	0.10 (p = .056)	-0.15 (p = .002)	-0.25 (p < .001)*

*p ≤ .001

References

Barzykowski, K., & Niedźwieńska, A. (2016). The Effects of Instruction on the Frequency and Characteristics of Involuntary Autobiographical Memories. *PLOS ONE, 11*(6), e0157121. https://doi.org/10.1371/journal.pone.0157121

Boyacioglu, I., & Akfirat, S. (2015). Development and psychometric properties of a new measure for memory phenomenology: The Autobiographical Memory Characteristics Questionnaire. *Memory, 23*(7), 1070–1092. https://doi.org/10.1080/09658211.2014.953960
